# Role of NLRP3 Inflammasome in Rheumatoid Arthritis

**DOI:** 10.3389/fimmu.2022.931690

**Published:** 2022-06-27

**Authors:** Hui Yin, Na Liu, Keshav Raj Sigdel, Lihua Duan

**Affiliations:** ^1^ Department of Rheumatology and Clinical Immunology, Jiangxi Provincial People’s Hospital, Medical College of Nanchang University, Nanchang, China; ^2^ Department of Rheumatology and Clinical Immunology, Jiangxi Provincial People’s Hospital, The First Affiliated Hospital of Nanchang Medical College, Nanchang, China; ^3^ Department of Internal Medicine, Patan Academy of Health Sciences, Kathmandu, Nepal

**Keywords:** inflammasome, NOD-like receptor protein 3 (NLRP3), rheumatoid arthritis, IL-1β, inflammation

## Abstract

Rheumatoid arthritis (RA) is a chronic inflammatory disease characterized by multi-articular, symmetrical and invasive arthritis resulting from immune system abnormalities involving T and B lymphocytes. Although significant progress has been made in the understanding of RA pathogenesis, the underlying mechanisms are not fully understood. Recent studies suggest that NLRP3 inflammasome, a regulator of inflammation, might play an important role in the development of RA. There have been increasing clinical and pre-clinical evidence showing the treatment of NLRP3/IL-1β in inflammatory diseases. To provide a foundation for the development of therapeutic strategies, we will briefly summarize the roles of NLRP3 inflammasome in RA and explore its potential clinical treatment.

## 1 Introduction

Rheumatoid arthritis (RA) is a chronic systemic autoimmune disease with a pathological basis in synovitis, manifesting as symmetric polyarticular invasive joint inflammation with extra-articular organ involvement, and patients with rheumatoid arthritis are characterized by positive serum rheumatoid factor and Anti-citrullinated protein antibodies (ACPAs) (1). The global average prevalence of RA ranges from 0.5% to 1.0% (2), with a higher prevalence among women and the elderly (1, 2). However, the pathogenesis of RA is not fully understood, which hinders the diagnosis and treatment of RA. In addition, the use of biologics has improved the condition of patients with RA while increasing the risk of infection (3, 4). Therefore, indepth study of the pathogenesis of rheumatoid arthritis is of greater significance for the development of new treatment strategies, but the pursuit of curative effect needs to pay attention to the safety of drugs (5). The pathogenesis of RA involves many immune cells and cytokines, including interleukin-1 (IL-1), interleukin-6 (IL-6), interleukin-18 (IL-18), and tumor necrosis factor (TNF) as the main pro-inflammatory cytokines, which induce inflammatory response and osteoarthritis injury (6). The NLRP3 inflammasome is a key source of IL-1 and IL-18, and accumulating evidence suggests that the inflammasome plays a role in the pathogenesis of rheumatic diseases (7). Here we discuss the current progress of the NLRP3 inflammasome in rheumatoid arthritis.

## 2 Overview of NLRP3 Inflammasome

The inflammasome, a group of multimeric protein complexes, identifies pathogen-associated molecular patterns (PAMPs) and damage-associated molecular patterns (DAMPs) by pathogen-recognition receptors (PRRs) to mediate host immune responses. The inflammasome is generally composed of a PRR, pro-caspase-1, and an adaptor protein that connects the PRR with pro-caspase-1 (8). The inflammasome promotes the maturation and secretion of IL-1β and IL-18 during natural immune defense by activating caspase-1, and also regulates adaptive immunity (7). In addition, inflammasome also mediates the caspase-1-dependent programmed cell apoptosis (pyroptosis), which induces cell death under inflammatory and stressed pathological conditions (9–11).

### 2.1 Classification of Inflammasomes and Activation of NLRP3 Inflammasome

The nucleotide-binding domain(NOD)–like receptor(NLR) protein consists of an N-terminal recruitment domain, a central nucleotide-binding domain that mediates oligomerization, and carboxy-terminal Leucine Rich Repeats (LRRs) (12–14). Depending on the existence of NLR protein, inflammasomes are divided into NLR family and non-NLR family. The former includes nucleotide‐binding domain leucine‐rich repeat pyrin domain containing 1(NLRP1), NOD-like receptor family pyrin domain containing 3 (NLRP3), and NLR-family CARD-containing protein 4 (NLRC4), while the latter includes Absent in Melanoma 2 (AIM2), pyrin inflammasomes (15). Based on the activation of caspase during inflammasome formation, inflammasomes containing caspase 1 are known as canonical inflammasomes, and complexes containing caspase 4, caspase 5, or caspase 11 are known as non-canonical inflammasomes. The caspase recruitment domain(CARD) motifs of caspases 4, 5, and 11 can directly bind to a portion of the intracellular lipopolysaccharide, leading to the activation of these caspases and the subsequent secretion of IL1 and IL18 (16–18). The NLRP3 inflammasome is the most studied inflammasome, and the NLRP3 contains three domains: pyrin domain(PYD), NACHT and LRR (19, 20). Activation of the NLRP3 inflammasome requires two steps (21–23): In the priming step (signal 1), PRRs such as toll-like receptors (TLRs) recognize PAMPs or DAMPs to activate the NF-κB signaling pathway and upregulate the expression of NLRP3 and pro-IL-1β. During the activation step (signal 2), in response to multiple stimuli such as reactive oxygen species (ROS), K^+^ efflux, ATP, lysosomal rupture, and bacterial or fungal components (24–27),NLRP3 binds to the adaptor protein ASC through the PYD domain and recruits pro-caspase-1 through the CARD domain to finally form the NLRP3 inflammasome (28). After the activation, caspase-1 will cleave pro-IL-1, pro-IL-18, and gasdermin D (GSDMD), leading to an inflammatory response and pyroptosis (29–32).

### 2.2 Regulation of the NLRP3 Inflammasome Activation

The NLRP3 inflammasome can be activated by a variety of stimuli such as transmembrane movement of ions, mitochondrial dysfunction, lysosomal rupture, and ROS. Hence, the regulation of its activation process is quite complex. Some potential regulatory mechanisms have been studied, such as the role of NIMA-related kinase 7 (NEK7), the regulation of autophagy/mTOR pathway, and changes in subcellular localization (27, 33, 34).

#### 2.2.1 Regulation of the Activation Step

NEK7 is involved in mitosis and plays a crucial role in the activation of the NLRP3 inflammasomes. Under the mediation of ROS and K^+^ efflux, NEK7 combines with the LRR domain of NLRP3, regulating NLRP3 inflammasome assembly as well as caspase-1 activation (35–37). A recent study showed that the stress granule protein DEAD-box helicase 3(DDX3X) can also contribute to NLRP3 activation by interacting with NLRP3 (38). Autophagy is a physiological process in which lysosomes degrade intracellular pathogens, damaged organelles, and proteins, contributing to host defense and cell homeostasis. It has been shown that the autophagosome directly encapsulates and degrades the NLRP3 inflammasome to prevent its activation (39, 40). Current studies have highlighted the emerging concept of organelles involved in NLRP3 activation. The NLRP3 inflammasome is composed of three different proteins, and recent studies have indicated that alterations in the subcellular localization of these molecules are also responsible for the assembly and activation of NLRP3 inflammasome. Activation of NLRP3 requires contributions from mitochondrial signals for priming, and the endoplasmic reticulum(ER), trans-Golgi network, cytoskeletal infrastructure, and microtubule-organizing center (MTOC) for transport and assembly (41). Mitochondria-associated ER membranes(MAM) and MTOC NLRP3 locates in the endoplasmic reticulum (ER) and cytosol, and NLRP3 in the endoplasmic reticulum binds to ASC on the adjacent mitochondria at the MAM upon various stimuli. NLRP3 relocates to the microtubule tissue center in the presence of microtubule affinity regulating kinase 4 (MARK4). When NLRP3 reaches the MTOC, NEK7 binds with NLRP3, and the inflammasome is assembled (42). In the absence of activation, NLRP3 localizes in the cytoplasm and endoplasmic reticulum but relocalizes in the mitochondria and Golgi apparatus after activation (43). Stimulation of NLRP3 destroys the trans Golgi network (TGN) and turns it into a dispersed TGN (dTGN). Furthermore, phosphatidylinositol-4-phosphate on dTGN promotes NLRP3 aggregation, which is essential for downstream ASC oligomerization and caspase-1 activation (44). Overall, these data suggest that spatially interaction among ER-mitochondria-Golgi apparatus is closely associated with NLRP3 activation, and understanding these regulatory processes may reveal new checkpoints for inflammasome.

A recent structural modeling study using cryo-electron microscopy described a double-ring cage structure held by 6-8 NLRP3 dimers *via* LRR-LRR interaction with the PYD shielded within the assembly to avoid premature activation. The cage structure may provide a mechanism for rapid activation of NLRP3 when NLRP3 monomers are assembled and in a standby state to sense signals and convert to their active conformation. They also confirmed that NLRP3 cage is also necessary for the dispersal of TGN. However, it is worth considering whether the conformation of cage is regulated by factors such as ion flux alteration and post-translational modifications (45). Moreover, Immunometabolism and circadian oscillation are also involved in regulating NLRP3 activation (46, 47).

#### 2.2.2 Regulation of the Priming Step

During the priming of NLRP3 inflammasome activation, NLRP3 protein expression is upregulated. And several mediators are involved in regulating NLRP3 transcription, including myeloid differentiation primary response 88 (MyD88) and TIR domain-containing adapter-inducing interferon-β (TRIF) (21), IL1 receptor-associated kinase (IRAK1) (48), Fas-associated protein with death domain (FADD) and caspase-8 (49). These mediators drive the transcription of the NLRP3 gene in preparation for activation. Additionally, it has been demonstrated that miRNAs regulate NLRP3 mRNA translation by binding to the untranslated regions (UTRs) of the transcript (50, 51). Post-translational modifications, such as ubiquitination and phosphorylation, are also important regulators of NLRP3 inflammasome activation. NLRP3 inflammasome ubiquitinates in macrophages when activated (52). It was reported that the vitamin D receptor negatively regulates NLRP3 activation by blocking deubiquitination (53). The process of ubiquitination is catalyzed by a series of enzymes, including E3 ubiquitin ligases. The E3 ligase tripartite motif-containing protein(TRIM31) has been reported to promote NLRP3 polyubiquitination thus inhibiting the excess activation of NLRP3 inflammasome (54). Dopamine inhibition of NLRP3 inflammasome is also achieved by the E3 ligase MARCH7-mediated ubiquitination of NLRP3 protein (55). However, another research showed that the E3 ligase Pellino2 promotes NLRP3 inflammasome activation through ubiquitination of NLRP3 (56). The exact mechanism by which multiple E3 ligases interact with different NLRP3 sites remains to be fully determined. Previous studies showed that NLRP3 is a substrate of protein kinase A (PKA), which phosphorylates NLRP3 at ser295 and inhibits the activation of the NLRP3 inflammasome (57, 58). However, protein kinase D plays a facilitating role in NLRP3 inflammasome activation at the same site (59). In addition, protein tyrosine phosphatase non-receptor type 22 (PTPN22) and phosphotase 2A (PP2A) can promote NLRP3 activation through dephosphorylation (60, 61). Epigenetic factors also regulate the activation of NLRP3 inflammasome, including DNA methylation and histone modifications. NLRP3 is methylated in health and mediates inflammatory suppression. Histone acetylation mediates the inflammatory response, while histone deacetylation induces inflammation resolution (62). It has been demonstrated that NLRP3 inflammasome expression can be down-regulated by inhibiting histone acetylation on NLRP3 promoter (63).

### 2.3 Immunomodulatory Effects of the NLRP3 Activation Products

The NLRP3 inflammasome is an important bridge connecting innate and adaptive immunity, and the activated NLRP3 inflammasome activates caspase-1 to produce biologically active IL-1 and IL-18. IL-1 and IL-18 belong to the IL-1 family and play a critical role in host immune regulation. IL-1 is a potent proinflammatory cytokine, mainly expressed on monocytes, macrophages and dendritic cells (DCs). IL-1β induces the upregulation of adhesion molecules and chemokines, leading to leukocytes recruitment and ultimately triggering a series of inflammatory responses (64). DC is an antigen-presenting cell that induces T cell activation (65). IL-1 activates DC to generate interferon-gamma (IFN-γ) in T cells. As a T cell co-stimulator, IL-1 induces T cell differentiation and polarization, especially toward T helper type 17 (Th17)cells. And IL-1β induces differentiation of naive CD4 + T cells into Th17 and also promotes Th9 differentiation in concert with other cytokines (66, 67). Moreover, IL-1β also facilitates the proliferation of B cells and the production of antibodies. IL-18, also known as IFN-γ inducing factor, drives the production of IFN-γ in Th1 cells and also works with IL-12 and IL-15 to activate natural killer (NK) cells to induce IFN-γ production (68). In epithelial cells, IL-18 regulates the function of Th17 cells and regulatory T(Treg) cells, resulting in an imbalance of Th17/Treg (69).As activation products of NLRP3 inflammasome, IL-1β and IL-18 contribute to proinflammatory T cell differentiation and activate adaptive immune responses (70). Accordingly, the NLRP3 inflammasome plays a vital role in immune regulation through leading to autoimmune diseases by its dysfunction or hyperactivation, and rheumatoid arthritis is one of them.

### 2.4 Role of IL-1β and IL18 in RA

The IL-1 family is the important inflammatory regulator that promotes the activation of innate immune system cells and is involved in the pathological process of various diseases. IL-1β, the best functionally member of the IL-1 family, is one of the major pathogenic factors of RA and mediates the destruction of bone and cartilage (71). Receptor activator of nuclear factor kappa-B ligand (RANKL)is the key osteoclastogenic cytokine that binds receptor activator of NF-kB(RANK) on osteoclast precursor cells and mediates osteoclast differentiation and activation, leading to bone resorption. IL-1β upregulates RANKL production, enhances its activity and stimulates osteoclast production to induce bone erosion (72). IL-1β also acts on osteoclast progenitors to stimulate osteoclastogenesis (73). In addition, IL-1β acts in concert with other inflammatory factors such as TNF-α to amplify the inflammatory response and induce bone loss (71). In rheumatoid arthritis synovium, IL1β is involved in cartilage degeneration by stimulating fibroblasts and chondrocytes to secrete matrix metalloproteinase (MMF), which in turn exacerbates synovial inflammation and bone destruction (73). Meanwhile, IL-1β impairs the synthesis of new bone matrix and inhibits osteoblast production in RA, thereby reducing new bone generation (74).

IL-18 is a pleiotropic cytokine that plays an important role in the flare and maintenance of the inflammatory response during RA. IL-18 plays a role by activating T cells in synovium to produce inflammatory cytokines, RANKL and so on, mediating bone destruction (75). Pannus formation is one of the pathological features of RA, and is also the main cause of joint lesions and cartilage destruction. *In vitro*, IL-18 triggers the production of vascular growth factors such as vascular endothelial growth factor, monocyte chemotactic protein 1, and stromal cell-derived factor 1, and facilitates the formation of vascular opacities (76).

## 3 The NLRP3 Inflammasome and RA

Rheumatoid arthritis (RA) is one of the most common autoimmune diseases. Increasing evidence suggests that the NLRP3 inflammasome is involved in the pathogenesis of RA. Anti-citrullinated protein antibodies are a group of autoantibodies against citrullinated proteins/peptides and are biomarkers of RA. ACPA promotes IL-1 production in rheumatoid arthritis by activating the NLRP3 inflammasome (77, 78). Several studies have shown an upregulation of NLRP3 mRNA and NLRP3-associated proteins in monocytes, macrophages, and dendritic cells in RA patients (79–81). Polymorphisms in the NLRP3 gene indirectly reflect the susceptibility, disease severity and treatment effect of RA. Rs4612666, rs10754558, rs10159239, and rs35829419 have been investigated as NLRP3 single nucleotide polymorphisms (SNPs) associated with susceptibility to rheumatoid arthritis, in which rs35829419 and CARD8 rs2043211 mutations are related to disease severity, while rs10159239 and rs4612666 are related to the therapeutic response of RA on anti-TNF (12, 82–85).

### 3.1 NLRP3 Inflammasome Mediates the Pathogenesis of RA

The involvement of the NLRP3 inflammasome in RA pathogenesis has been demonstrated in both animal and cell experiments. Genetics is closely linked to susceptibility to RA (86). The RA susceptibility gene A20, also called tumor necrosis factor alpha-induced protein 3 gene (TNFAIP3), is a cytokine-inducible protein that inhibits apoptosis and activates NF-κB. A20-deficient mice were prone to spontaneous erosive arthritis, which was found to be related to elevated NLRP3 expression and IL-1β secretion (87). Collagen-induced arthritis (CIA) is the most commonly used animal model to support the role of inflammasome in inflammatory arthritis (88). The expression of NLRP3 was proved to be positively correlated with arthritis severity in the synovium of CIA mice (89). Likewise, antigen-induced arthritis (AIA) mice exhibit severe joint inflammation with increased expression of IL-1β and NLRP3 inflammasome in their synovium (90). In CFA-induced arthritic rats, cinnamaldehyde alleviates the inflammatory response by activating succinate/hypoxia-inducible factor-1 (HIF-1) to suppress NLRP3-derived IL-1β (91). These data indicate that NLRP3 inflammasome is involved in the pathogenesis of RA. Activation of inflammasome can also occur in non-phagocytes, such as T cells, endothelial cells, and epithelial cells. Th17 cells mediate pro-inflammatory responses through the secretion of IL-17A and TNF-α, leading to tissue destruction, articular cartilage and bone damage. In contrast, Tregs mediate anti-inflammatory responses through the secretion of IL-10 and TGF-β. Recent studies have noted that the imbalance of Treg/Th17 cells affects the inflammatory response in RA (92). It is known that tofacitinib also restores the balance of Treg/Th17 cells in rheumatoid arthritis and alleviates the inflammatory response by inhibiting NLRP3 inflammasome (93). Furthermore, elevated extracellular Ca^2+^ concentration promotes the uptake of colloidal calciprotein particles (CPPs) by monocytes in RA patients and contributes to NLRP3 inflammasome activation (94). The above studies showed NLRP3 inflammasome is involved in RA by regulating different cells.

### 3.2 NLRP3 Inflammasome as a Protective Factor in RA

However, neutrophils were less sensitive to pyroptosis compared with macrophages (95, 96), and expression of NLRP3 and ASC were significantly decreased in neutrophils of RA patients. Meanwhile, mRNA expression of NLRP3 in neutrophils was negatively correlated with the 28-joint Disease Activity Score based on C-reactive protein (DAS28-CRP) in patients with RA (97, 98). These results indicate that different cell types exhibit different responses to inflammasome stimulation, and NLRP3 inflammasome may be protective in RA. The conclusion was also confirmed in another research (99). The pathogenic role of NLRP3 has been demonstrated in systemic lupus erythematosus, inflammatory bowel disease, and type 1 diabetes mellitus(T1DM). Nevertheless, the protective effect of NLRP3 inflammasome has also been illustrated in these diseases (34). The exact mechanism of this effect is not specified, requiring a deeper understanding of the role of NLRP3 inflammasome in diseases. But it may be explained by the following speculations: a)NLRP3 performs different functions at different stages of the disease (**Table1**) (102); b) NLRP3 exerts opposite effects through the regulation of other cytokines (104); c) NLRP3 maintains immune homeostasis and thus plays a protective role. Recent studies showed that NLRP3 inflammasome is a crucial regulator of intestinal homeostasis. NLRP3 inflammasome supports the integrity of the intestinal mucosal barrier and also has a regulatory impact on intestinal flora (105, 106).

**Table 1:** Role of NLRP3 inflammasome in different stages of different diseases.

Disease	Role of NLRP3 inflammasome or IL-1 and IL-18	Reference
IAV infection	NLRP3 inflammasome mediates protective immune defense in the early stage while exacerbates inflammatory responses in the late stage of the disease.	(100)
CAPS	The inflammatory effects of IL-18 were significantly enhanced in young mice compared with aged mice.	(101)
T1DM	NLRP3 inflammasome acts as a protective factor in the early stages of the disease.	(102)
Cholestatic liver injury	NLRP3 inflammasome plays a protective role in acute cholestatic liver injury while mediates the pathogenic role in chronic cholestatic liver injury.	(103)

IAV, influenza A virus; CAPS, cryopyrin-associated periodic syndromes; T1DM: Type 1 diabetes mellitus.

## 4 Potential Therapeutic Targets for the NLRP3 Inflammasome in Rheumatoid Arthritis

### 4.1 Blockade of NLRP3 Activation Products

Targeted treatment of rheumatoid arthritis by blocking cytokines has been recognized. Anakinra is a recombinant form of a human IL-1 receptor antagonist that competitively inhibits IL-1αand IL-1β (107). Although approved by the Food & Drug Administration (FDA) for some patients with rheumatoid arthritis (108), Anakinra is only moderately effective and is inferior to TNF-α inhibitors, indicating its poor applicability (109). Additionally, a human monoclonal IL-1β antibody canakinumab, and a decoy receptor of IL-1α and IL-1β rilonacept have been focused on in rheumatoid arthritis (27). Inhibition of caspase-1 blocks the downstream signaling of inflammasome and the release of active IL-1β, thereby resolving the inflammation. The most studied caspase-1 inhibitors are VX-765 (belnacasan) and VX-740 (pralnacasan).VX-740 attenuated the joint damage in both RA and OA mice, but its application was limited by animal hepatotoxicity (110, 111). VX-765 is structurally similar to VX-740, acting by covalent modification of the catalytic cysteine residues in the caspase-1 active site, which has been tested in phase II trials for psoriasis and epilepsy, but not yet in rheumatoid arthritis (112, 113). Non-steroidal anti-inflammatory drugs (NSAIDs) are universally used and their anti-inflammatory property is the inhibition of cyclooxygenase (COX) isozymes. It has been proposed that inhibition of caspase enzymes reduces the production of proinflammatory cytokines, suggesting that there may be other targets for NSAIDs to promote anti-inflammatory responses beyond the COX pathway (114).

### 4.2 Inhibition of NLRP3 Inflammasome Activation

Inhibition of the NLRP3 inflammasome includes the blockage of the NLRP3 inflammasome assembly and the suppression of the NLRP3-related signaling pathways (115).

#### 4.2.1 Inhibition of NLRP3 Inflammasome Assembly

Compound MCC950 is the most widely studied inhibitor of NLRP3 inflammasome with high efficiency and specificity. MCC950 ameliorates rheumatoid arthritis injury by inhibiting NLRP3 activation and subsequent IL-1β production (79). It has been reported that MCC950 blocks ATP hydrolysis and suppresses NLRP3 inflammasome formation and activation by directly interacting with the NACHT domain of NLRP3 (116, 117). However, the application of MCC950 was limited by hepatotoxicity in a phase II clinical trial of RA (27). Both CY-09 and tranilast (TR) (118–120) bind directly to the ATP-binding motif of the NACHT domain of NLRP3 to inhibit ATPase activity and thus prevent the assembly of NLRP3 inflammasome, which may be promising in the management of rheumatoid arthritis. RRX-001 is a highly selective NLRP3 inflammasome inhibitor that acts by covalently binding to cysteine 409 of NLRP3 to interfere with its assembly. It is a novel NLRP3-related disease therapeutic target identified in recent years (121). However, RRX001 contains high-energy nitro functional groups, which may cause drug toxicity. Compound 149-01, an analog of RRX001 without high-energy nitro functional groups, was recently identified as a potent and specific NLRP3 inhibitor that inhibits NLRP3 activation and attenuates inflammatory responses *in vivo* and vitro by preventing NLRP3 from binding to NEK7 (122). Licochalcone B (LicoB), a major component of traditional medicinal herb licorice, was also found to disturb the interaction between NLRP3 and NEK7, thus inhibiting NLRP3 activation (123). LL-Z1640-2(LLZ) is a preclinical drug that primarily targets TGF-β-activated kinase-1(TAK1), which produces a variety of proinflammatory cytokines and inflammatory mediators in RA. LLZ showed superior therapeutic efficacy against RA in preclinical trials. In CIA mice, LLZ was observed to significantly prevent the formation and activation of NLRP3 inflammasome in synovial macrophages and osteoclasts (124). A recently identified novel compound 59 known as J114 was shown to interfere with NLRP3-ASC interaction and potently suppress ASC oligomerization during NLRP3 activation. Interestingly, the function of J114 exhibits species differences (125). Although research on this compound is still in the very beginning stage, J114 may be useful for further exploration of the exact modulatory mechanism of NLRP3 inflammasome.

#### 4.2.2 Inhibition of NLRP3 Inflammasome Related Signaling Pathway

As previously mentioned, the priming and activation of the NLRP3 inflammasome are regulated by multiple signaling pathways, and a number of NLRP3 inflammasome inhibitors have been developed to target these regulators. For example, taraxasterol and parthenolide suppress the activation of NLRP3 inflammasome by blocking the activation of NF-κB (126, 127), and celastrol resolves the inflammatory response to RA through inhibition of the ROS/NF-κB/NLRP3 axis (128). In addition, β-arrestins are vital regulators of G protein-coupled receptors (GPCRs), and β-arrestin-2(βArr2) has anti-inflammatory effects in a variety of inflammation-related diseases. Current study demonstrated that βArr2 effectively alleviates joint inflammation by inhibiting the NF-κB/NLRP3 signaling pathway in CIA mice (129). The traditional disease-modifying anti-rheumatic drug hydroxychloroquine is widely recognized in RA treatment. Hydroxychloroquine (HCQ) inhibits Ca^2+^-activated K^+^Channels, resulting in an impaired NLRP3 inflammasome activation (130). The mammalian target of rapamycin (mTOR) which regulates cellular metabolism and plays a negative role in regulating autophagy, is a serine-threonine protein kinase and belongs to the phosphatidylinositol 3-kinase-related kinase (PIKK) family. As an mTOR inhibitor, rapamycin treats RA by inducing autophagy to inhibit NLRP3 inflammasome and inflammation (131).

## Discussion

NLRP3 inflammasome-driven inflammation accompanies the pathogenesis of autoimmune diseases which includes rheumatoid arthritis and makes NLRP3 inflammasome an attractive drug target. Meanwhile, NLRP3-mediated immune responses are critical for host defense against bacteria, viruses, and fungi (132–134). Therefore, a balance between activators and inactivators of NLRP3 inflammasomes are required to maintain immune homeostasis. Compared with cytokine blockade, molecules directly targeting NLRP3 inflammasome are more advantageous (135, 136), but there are no clinically available therapeutic agents now. Given the important role of NLRP3 inflammasome in both innate and adaptive immunity, the development of NLRP3 inhibitory drugs should be handled with caution. Many inhibitors of NLRP3 inflammasome have been identified, and several small molecule compounds are in clinical trials. Further elucidation of the clinical efficacy and safety of these inhibitors is still needed.

## Summary

Inflammasomes have become the focus of research in the field of inflammatory diseases. Increasing evidence suggests that the NLPR3 inflammasome plays a key role in the pathogenesis of rheumatic diseases. Excessive inhibition or activation leads to immune disorders that require precise regulation during NLPR3 inflammasome activation. Therefore, it is necessary to understand the mechanism of the NLPR3 inflammasome to explore promising therapeutic strategies for autoimmune diseases such as RA. Cytokines inhibitors remain limited due to infection risk, whereas NLRP3 inflammasome inhibitors show the best anti-inflammatory effects in animal models. The side effects of drugs may be unavoidable, while drug structure optimization is expected to break this dilemma and provide a practical and effective way for the treatment of RA.

## Author Contributions

HY and NL reviewed the literature and wrote the first draft. HY, NL, and LD reviewed the literature and finalized the manuscript. HY, YL, and LD revised the manuscript. All authors have read and approved the final manuscript.

## Funding

This work was supported by the National Natural Science Foundation of China (81960296 and 81871286), JiangXi Provincial Natural Science Foundation of China (20192ACB21006), Interdisciplinary Innovation Team, Frontier Science Key Research Project of Jiangxi Provincial People’s Hospital (19-008), Long-term (Youth) Project for Leading Innovative Talents in Jiangxi Province (Lihua Duan), Jiangxi Provincial Clinical Research Center for Rheumatic and Immunologic Diseases (20192BCD42005), Jiangxi Province Medical Leading Discipline Construction Project (Rheumatology), and Provincial and municipal joint construction projects of medical disciplines in Jiangxi Province (Rheumatology).

## Conflict of Interest

The authors declare that the research was conducted in the absence of any commercial or financial relationships that could be construed as a potential conflict of interest.

## Publisher’s Note

All claims expressed in this article are solely those of the authors and do not necessarily represent those of their affiliated organizations, or those of the publisher, the editors and the reviewers. Any product that may be evaluated in this article, or claim that may be made by its manufacturer, is not guaranteed or endorsed by the publisher.
