# Corrigendum: Role of NLRP3 inflammasome in rheumatoid arthritis

**DOI:** 10.3389/fimmu.2023.1228176

**Published:** 2023-07-18

**Authors:** Hui Yin, Na Liu, Keshav Raj Sigdel, Lihua Duan

**Affiliations:** ^1^Department of Rheumatology and Clinical Immunology, Jiangxi Provincial People’s Hospital, Medical College of Nanchang University, Nanchang, China; ^2^Department of Rheumatology and Clinical Immunology, Jiangxi Provincial People’s Hospital, The First Affiliated Hospital of Nanchang Medical College, Nanchang, China; ^3^Department of Internal Medicine, Patan Academy of Health Sciences, Kathmandu, Nepal

**Keywords:** inflammasome, NOD-like receptor protein 3 (NLRP3), rheumatoid arthritis, IL-1β, inflammation

In the published article, there was an error in **Table 1** as published. The role of IL-18 in CAPS was displayed as “The inflammatory effects of IL-1 and IL-18 were significantly enhanced in aged mice compared with young mice”. The correct statement is “The inflammatory effects of IL-18 were significantly enhanced in young mice compared with aged mice”. The corrected **Table 1** and its caption “Role of NLRP3 inflammasome in different stages of different diseases” appear below.

**Table 1:** Role of NLRP3 inflammasome in different stages of different diseases.

Disease	Role of NLRP3 inflammasome or IL-18	Reference
IAV infection	NLRP3 inflammasome mediates protective immune defense in the early stage while exacerbates inflammatory responses in the late stage of the disease.	(100)
CAPS	The inflammatory effects of IL-18 were significantly enhanced in young mice compared with aged mice.	(101)
T1DM	NLRP3 inflammasome acts as a protective factor in the early stages of the disease.	(102)
Cholestatic liver injury	NLRP3 inflammasome plays a protective role in acute cholestatic liver injury while mediates the pathogenic role in chronic cholestatic liver injury.	(103)

IAV, influenza A virus; CAPS, cryopyrin-associated periodic syndromes; T1DM: Type 1 diabetes mellitus.

In the published article, the reference in the sentence “And IL-1β induces differentiation of naive CD4 + T cells into Th17 and also promotes Th9 differentiation in concert with other cytokines. (67)” was incorrectly written as “(!!! INVALID CITATION!!! [58; 59])”. It should be “G. Xue, G. Jin, J. Fang, and Y. Lu, IL-4 together with IL-1β induces antitumor Th9 cell differentiation in the absence of TGF-β signaling. Nat Commun 10 (2019) 1376.” and “E.V. Acosta-Rodriguez, G. Napolitani, A. Lanzavecchia, and F. Sallusto, Interleukins 1beta and 6 but not transforming growth factor-beta are essential for the differentiation of interleukin 17-producing human T helper cells. Nat Immunol 8 (2007) 942-9”.

The reference in the sentence “Recent studies have noted that the imbalance of Treg/Th17 cells affects the inflammatory response in RA.(93)” was incorrectly written as “(!!! INVALID CITATION!!! [77])”. It should be “S. Jin, S. Sun, H. Ling, J. Ma, X. Zhang, Z. Xie, N. Zhan, W. Zheng, M. Li, Y. Qin, H. Zhao, Y. Chen, X. Yang, and J. Wang, Protectin DX restores Treg/T(h)17 cell balance in rheumatoid arthritis by inhibiting NLRP3 inflammasome via miR-20a. Cell Death Dis 12 (2021) 280.”

In the published article, the reference in the sentence “For example, taraxasterol and parthenolide suppress the activation of NLRP3 inflammasome by blocking the activation of NF-κB (127)” was incorrectly written as “(!!! INVALID CITATION!!! [103;104])”. It should be “J. Chen, W. Wu, M. Zhang, and C. Chen, Taraxasterol suppresses inflammation in IL-1β-induced rheumatoid arthritis fibroblast-like synoviocytes and rheumatoid arthritis progression in mice. Int Immunopharmacol 70 (2019) 274-283.” and “N. Li, Y. Wang, X. Wang, N. Sun, and Y.H. Gong, Pathway network of pyroptosis and its potential inhibitors in acute kidney injury. Pharmacol Res 175 (2022) 106033.”

In the published article, reference 52 (Z. Wang, Y. Kun, Z. Lei, W. Dawei, P. Lin, and W. Jibo, LncRNA MIAT downregulates IL-1β, TNF-ɑ to suppress macrophage inflammation but is suppressed by ATP-induced NLRP3 inflammasome activation. Cell Cycle 20 (2021) 194-203.) was incorrectly cited. It should be removed.

In the published article, reference 72 (X. Lu, L. Gilbert, X. He, J. Rubin, and M.S. Nanes, Transcriptional regulation of the osterix (Osx, Sp7) promoter by tumor necrosis factor identifies disparate effects of mitogen-activated protein kinase and NF kappa B pathways. J Biol Chem 281 (2006) 6297-306.) was incorrectly cited. It should be removed.

In the published article, there was an error. “NLRP” was wrongly written in a section title instead of “NLRP3”.

A correction has been made to **Section 3.2**, *NLRP inflammasome as a protective factor in RA*. This sentence previously stated:

“*NLRP inflammasome as a protective factor in RA*”

The corrected sentence appears below:

“*NLRP3 inflammasome as a protective factor in RA*”

The authors apologize for these errors and state that this does not change the scientific conclusions of the article in any way. The original article has been updated.

